# Influence of different preoperative fasting times on women and neonates in cesarean section: a retrospective analysis

**DOI:** 10.1186/s12884-019-2254-2

**Published:** 2019-03-29

**Authors:** Yi Li, Danchen Su, Yijuan Sun, Zurong Hu, Zaomei Wei, Jie Jia

**Affiliations:** 1 Department of Gynecology, Guangdong Women and Children Hospital, Guangzhou, China; 2 Department of Anesthesiology, Guangdong Women and Children Hospital, No.13 Guangyuan West Road, Guangzhou, 510010 China

**Keywords:** Preoperative fasting times, Cesarean section, Women, Neonates

## Abstract

**Background:**

This study was to evaluate the impact of different preoperative fasting conditions on women and neonates through a retrospective analysis.

**Methods:**

A total of 1599 women were divided into 5 groups according to different preoperative fasting times: group A: solid food ≥8 h; clear fluids ≥6 h; B: solid food ≥8 h; clear fluids ≥2 h < 6 h; C: solid food ≥6 h < 8 h; clear fluids < 2 h; D: solid food ≥2 h < 6 h; clear fluids < 2 h; E: solid food < 2 h; clear fluids < 2 h.

**Results:**

Incidence rate of vomiting of women was lower in group C (solid food ≥6 h < 8 h and clear fluids < 2 h) than other groups (*P* <  0.05). Compared with the fasting for a long time (groups A and B: solid food > 8 h and clear fluids > 2 h at least), the incidence rate of hypoglycemia and acidosis of neonates in group C displayed a certain decrease (P <  0.05). Although shorter fasting times (solid food < 6 h at least) reduced the incidence of hypoglycemia and acidosis in neonates, it increased the risk of vomiting of women.

**Conclusion:**

The preoperative fasting of solid food ≥6 h < 8 h and clear fluids < 2 h reduces the incidence of vomiting in women’s anesthesia and the risk of hypoglycemia and acidosis in neonates.

## Background

The increase in incidence of caesarean sections worldwide is associated with multiple factors, however evidence suggests that a considerable proportion continue to be driven by convenience without an evidence based/medical indication for the procedure [1]. The current recommendation of the World Health Organization (WHO) for cesarean section (section C) is that this clinical practice should only be used when the health or life of women or neonate is threatened [2]. However, caesarean section does not mean that there is no maternal-infant risk and requires a cautious medical assessment to determine if its risk exceeds benefits. The occurrence of vomiting and regurgitation and aspiration has been considered as important problems that need to be focused on during and after caesarean section [3, 4]. Multiple pregnancies, polyhydramnios, obesity, lithotomy position, pressure of the bottom of the uterus, prenatal feeding also will further increase the risk of vomiting and regurgitation and aspiration [2, 5, 6]. And, women undergoing caesarean section are often given general anesthesia for surgery, so aspiration after regurgitation and/or vomiting may cause aspiration pneumonia, respiratory failure or acute respiratory distress syndrome, and even death [3, 4, 7].

For clinical safety, prior to anesthesia induction and/or surgery, the patients or recipients are required to fast all food and liquids within the prescribed time and the preoperative fasting intervention has been showed to ensure physiological stability and reduce the intraoperative and postoperative complications [8]. To a woman who has to accept caesarean section, preoperative fasting often is required. But given the diversity and complexity of maternal and fetal conditions, established fasting standards are often not strictly carried out. In addition, a long time of fasting before cesarean section may bring some adverse effects on women or neonates. We retrospectively analyzed the data of different preoperative fasting conditions of women before caesarean section in our hospital within 2 years in order to determine the potential impact of different fasting conditions on the health of women and neonates.

## Methods

### Inclusion criteria

Inclusion criterion: (1) pre-anesthetic assessment of the women must be performed using the American Academy of Anesthesiologists (ASA) grading system [9]; (2) clinical medical records of the women must be complete; (3) full-term childbirth (more than 37 weeks of pregnancy); (4) meeting the indications of cesarean section operation according to the criterion made by Obstetrics group, Obstetrics and Gynecology, Chinese Medical Association [10]; (5) receiving the same anesthetic method (epidural block); and (6) without mental illness, serious heart, lungs, liver, kidney and blood system diseases.

### Exclusion criteria

Exclusion criterion: (1) women combined with serious surgical diseases; (2) placenta accreta and placenta adhesion; (3) twins and multiple births; (4) esophageal hiatal hernia and gastroesophageal reflux disease history; (5) fetal distress; (6) anemia, pregnancy-induced hypertension, abnormal glucose metabolism, and others with pregnancy complications, and (8) using gastrointestinal stimulation drugs, antacids, anticholinergic drugs before surgery.

### Data collection

A total of 1599 full-term women (more than 37 weeks of pregnancy) who received the cesarean section at the Guangdong Women and Children Hospital, Guangzhou, China from January 2015 to December 2017 were included in this retrospective study. The following data on women were extracted: age (years), frequency of delivery (times), body mass index (BMI) (kg/m^2^), the situation of vomiting (observation time is from the beginning of anesthesia to the end of surgery and recorded by anesthesiologists), blood glucose, electrolyte disturbance and abdominal distension. The data from neonates: birth weight (kg), gender (boys or girls), macrosomia (≥4000 g) (yes or no), small for gestationalage or large for gestationalage.

### Anesthetic methods

Considering that the anesthetic results may affect some physiological conditions of women or neonates, the women included this study received the same anesthetic method (epidural block) [11]. The anesthetic effects were evaluated from three aspects of analgesia, muscle loosening effect and visceral traction reflex [11], which were divided into 4 levels (Level 1: poor analgesic effect, intense pain, tight abdominal muscles, serious traction reaction; Level 2: general analgesic effect, mild pain, abdominal muscle tension, slight traction reaction; Level 3: good analgesic effect, slight abdominal muscle tension, no traction reaction; Level 4: excellent analgesic effect, loose abdominal muscles, no traction reaction).

### Fasting condition grouping

According to the time of preoperative fasting, fasting conditions were divided into 5 groups: A, solid food ≥8 h and clear fluids ≥6 h; B, solid food ≥8 h and clear fluids ≥2 h < 6 h; C, solid food ≥6 h < 8 h and clear fluids < 2 h; D, solid food ≥2 h < 6 h and clear fluids < 2 h; E, solid food < 2 h and clear fluids < 2 h.

### Test of blood glucose level

Within 10 min after the birth, the heel peripheral blood of neonate and elbow vein blood of women was collected. The blood samples were sent to test as soon as possible through the Siemens Automatic Biochemical Analyzer using a glucose oxidase method. Glucose test kit was purchased from Shanghai Shenergy-Diasys Diagnostic Technology Co., Ltd. The blood glucose level of neonates was defined as hypoglycemia when it was less than 2.6 mmol/L [12]. The women with blood glucose levels less than 3.9 mmol/L considered to be hypoglycemic.

### Test of potential of hydrogen (pH) in arterial blood

After the birth, the umbilical arterial blood of neonates immediately collected and was sent to test as soon as possible by GEM Premier 3000 Arterial Blood Gas Analyzer (American Experimental Equipment Company) using an electrode method. The GEM PAK blood gas detection kit (product standard: YZB/USA 0153–2012) was purchased from Jinan Raddchen Technology Trading co., LTD.

### Apgar scores

The clinical state of neonates was evaluated using the Apgar scores system, which based on five physical signs (heart rate, respiratory effort, reflex irritability, muscle tone and colour) presented shortly after birth. The value of Apgar scores range from zero to 10. Scores 7 and above are generally normal, 4 to 6 fairly low, and 3 and below are generally regarded as critically low [13].

### Statistical analysis

The statistical ideas used in this study are as follows: (1) The measurement data of the normal distribution for comparison between groups, such as the birth weight of the newborn, the gestational age, etc., were expressed with mean ± standard deviation and analyzed by Student’s T test and variance analysis; (2) The non-normal distribution data, blood glucose and value of pH, were compared with non-parametric test; (3) The counting data, such as postoperative complications in women and general clinical data for neonates, were expressed in terms of rate (%) and a chi-square test was used for comparison between groups; (4) Multifactor logistic regression analysis was used to analyze the relative risk factors on the incidence of hypoglycemia and low-pH value caused by different fasting conditions, (5) SPSS 21.0 statistical software (SPSS Institute, Chicago, USA) was used to analyze the data. *P* < 0.05 was considered to be statistically significant.

## Results

### Clinical features of women showed a better comparability

**Fig. 1:**
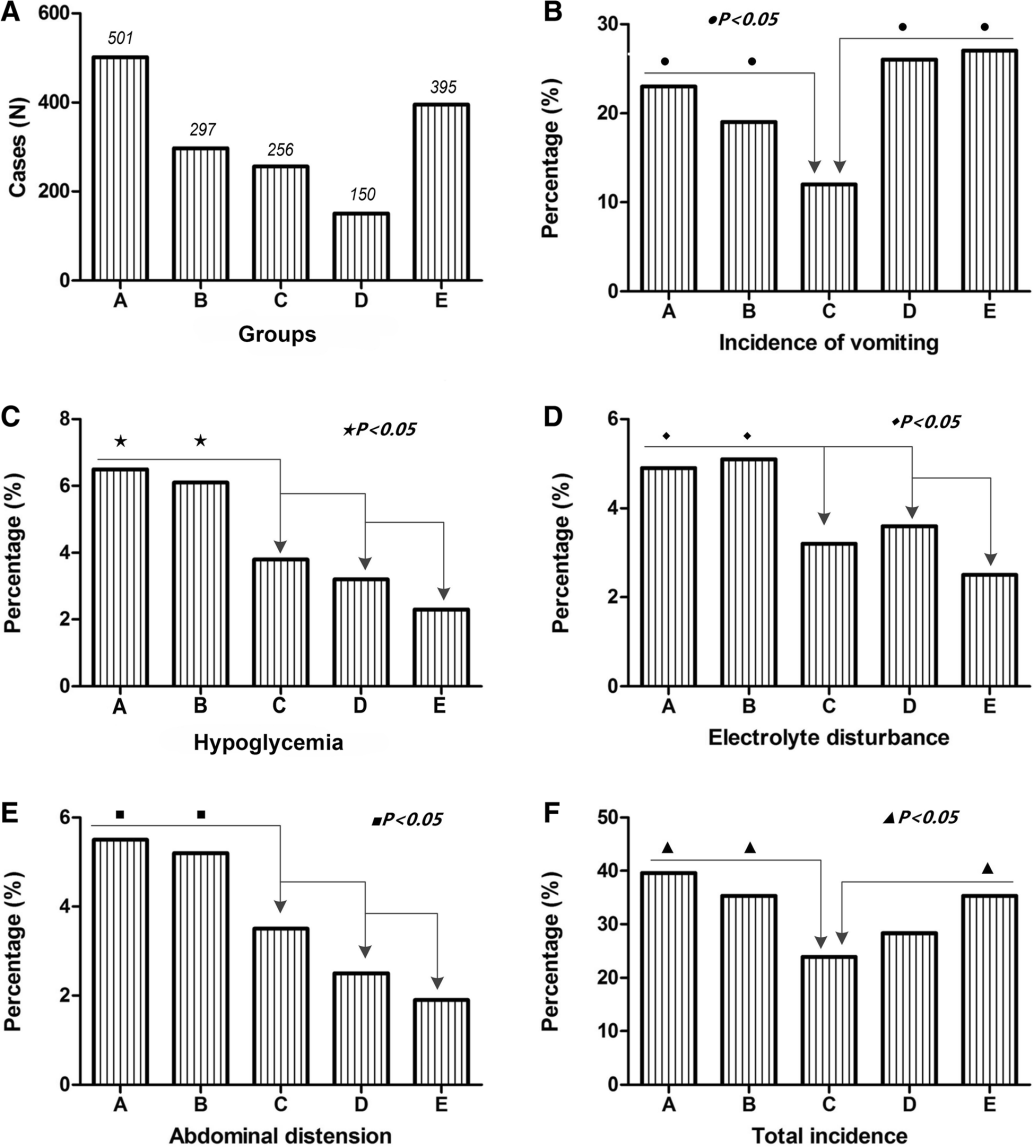
Influences of different fasting conditions on complications of women. **a**. The proportion of women distributed in the five groups, group A and E had relatively more cases; **b**. ^●^, the incidence of vomiting in groups A, B, D and E was significantly higher than that in group C (*p* < 0.05); **c**. ^★^, the incidence of hypoglycemia in groups A and B was significantly higher than that in group C, D and E (*p* < 0.05); **d**. ^◆^, the incidence of electrolyte imbalance in groups A and B was significantly higher than that in group C, D and E (*p* < 0.05); **e**. ^■^, the incidence of abdominal distension in groups A and B was significantly higher than that in group C, D and E (*p* < 0.05); **f**. ^▲^, the total incidence rate of complications in groups A, B and D were higher than that in group C and E (*p* < 0.05). Notes:A, solid food ≥8 h and clear fluids ≥6 h; B, solid food ≥8 h and clear fluids ≥2 h < 6 h; C, solid food ≥6 h < 8 h and clear fluids < 2 h; D, solid food ≥2 h < 6 h and clear fluids < 2 h; E, solid food < 2 h and clear fluids < 2 h

As shown in Table 1, a total of 1599 women were divided into 5 groups according to the time of preoperative fasting (Fig. 1a) and there was no difference in age distribution among the groups (*P* > 0.05). There were no statistical differences between groups, regardless of narcotic effect (*P* > 0.05), frequency of delivery (*P* > 0.05), and body mass index (BMI) (*P* > 0.05). Moreover, the proportions of overweight and obese among groups were not statistically different (*P* > 0.05).

**Table 1:** Comparison of clinical data on women

Groups	Items	Cases	Ages (Years)	Narcotic effect (Levels)	Frequency of delivery (Times)	BMI (kg/m^2^)	Overweight or obese (Cases)
	Fasting period	(N)	(M ± SD)	Mean (Scope)	(Scope)	(M ± SD)	(%)
A	Solid food ≥8 h; clear fluids ≥6 h	501	31.3 ± 4.1	3~4	0~2	21.3 ± 4.5	55 (11.0%)
B	Solid food ≥8 h; clear fluids ≥2 h < 6 h	297	31.0 ± 4.4	3~4	0~2	21.6 ± 4.2	35 (11.8%)
C	Solid food ≥6 h < 8 h; clear fluids < 2 h	256	30.3 ± 3.9	3~4	0~2	20.9 ± 5.1	27 (10.7%)
D	Solid food ≥2 h < 6 h; clear fluids < 2 h	150	32.0 ± 4.5	3~4	0~2	21.7 ± 4.0	18 (12.1%)
E	Solid food < 2 h; clear fluids < 2 h	395	31.1 ± 4.1	3~4	0~1	21.9 ± 4.8	49 (12.5%)
	Statistical value		0.055	0.188	2.568	0.465	0.989
	*P* value		0.883	0.665	0.245	0.089	0.560

*N* number, *BMI* body mass index, *M ± SD* mean ± standard deviation

### Impact of fasting conditions on women themselves

As shown in Table 2, the incidence rates of vomiting of women in groups A (solid food ≥8 h and clear fluids ≥6 h), B (solid food ≥8 h and clear fluids ≥2 h < 6 h), D (solid food ≥2 h < 6 h and clear fluids < 2 h) and E (solid food < 2 h and clear fluids < 2 h) were 23, 19, 25.8 and 26.8%, respectively, which was significantly higher than 11.6% in group C (solid food ≥6 h < 8 h and clear fluids < 2 h) (*P* < 0.05) (Fig. 1b). Compared with groups C, D and E, the incidence of hypoglycemia (A, 6.5%; B, 6.1%) (*P* < 0.05) (Fig. 1c), electrolyte imbalance (A, 4.9%; B, 5.1%) (*P* < 0.05) (Fig. 1d) and abdominal distension (A, 5.5%; B, 5.2%) (*P* < 0.05) (Fig. 1e) of women in groups A and B all obviously went up (*P* < 0.05). The total incidence rate of complications in group C was lower than other groups (*P* < 0.05) (Fig. 1f).

**Table 2:** Comparison of postoperative complications in women

Groups	Items	Cases	Incidence of vomiting	Hypoglycemia	Electrolyte disturbance	Abdominal distension	Total incidence
	Fasting period	(N)	(%)	(%)	(%)	(%)	(%)
A	Solid food ≥8 h; clear fluids ≥6 h	501	115 (23.0)^●^	33 (6.5)^★^	25 (4.9)^◆^	28 (5.5)^■^	198 (39.6)^▲^
B	Solid food ≥8 h; clear fluids ≥2 h < 6 h	297	56 (19.0)^●^	18 (6.1)^★^	15 (5.1)^◆^	15 (5.2)^■^	105 (35.3)^▲^
C	Solid food ≥6 h < 8 h; clear fluids < 2 h	256	30 (11.6)	10 (3.8)	8 (3.2)	9 (3.5)	61 (23.9)
D	Solid food ≥2 h < 6 h; clear fluids < 2 h	150	39 (25.8)^●^	5 (3.2)	5 (3.3)	4 (2.7)	42 (28)
E	Solid food < 2 h; clear fluids < 2 h	395	106 (26.8)^●^	9 (2.3)	10 (2.5)	8 (1.9)	139 (35.3)^▲^
	*P* value		< 0.05	< 0.05	< 0.05	< 0.05	< 0.05

*N* number, *BMI* body mass index, *M ± SD* mean ± standard deviation; ^●^, the incidence of vomiting in groups A, B, D and E was significantly higher than that in group C; ^★^, the incidence of hypoglycemia in groups A and B was significantly higher than that in group C, D and E; ^◆^, the incidence of electrolyte disturbance in groups A and B was significantly higher than that in group C, D and E; ^■^, the incidence of abdominal distension in groups A and B was significantly higher than that in group C, D and E; ^▲^, the incidence of total complications in groups C was significantly lower than that in group A, B and E

### Clinical features of neonates showed a better comparability

**Fig. 2:**
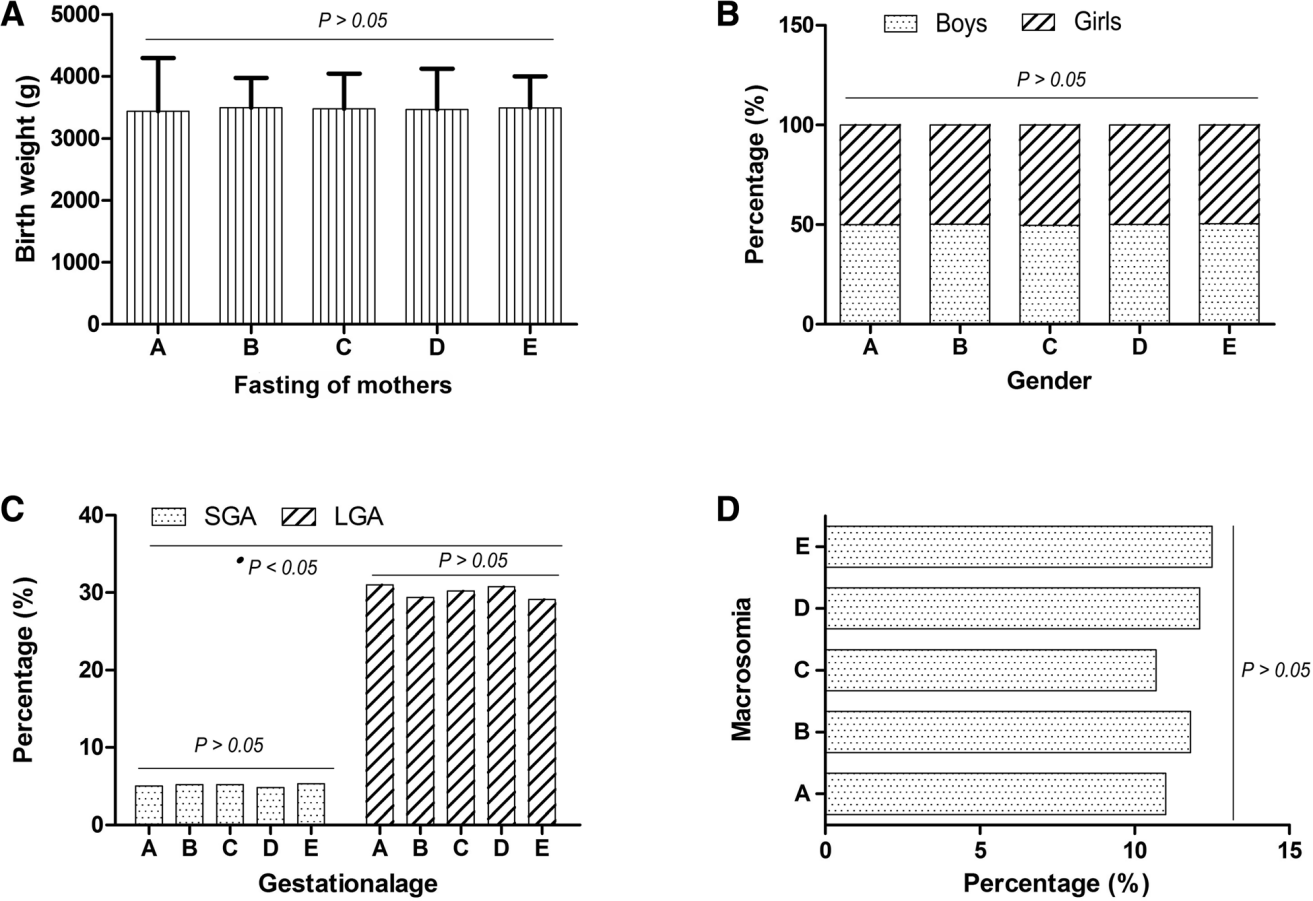
Comparison of clinical data for neonates. **a**. The birth weight of neonates distributed in the five groups showed no statistical difference (*P* = 0.183); **b**. There was no statistically difference between the number of boys and girls in the five groups (*P* > 0.5). **c**. In terms of gestational age, neither for small gestationalage (SGA) (*P* = 0.759) nor for large gestationalage (LGA) (*P* = 0.791) showed significant differences between the five groups. However, the proportion of LGA was much higher than that of SGA in total neonates (^●^*p* < 0.05); **d**. The incidence of macrosomia was not significantly different between the five groups (P > 0.5). Notes:A, solid food ≥8 h and clear fluids ≥6 h; B, solid food ≥8 h and clear fluids ≥2 h < 6 h; C, solid food ≥6 h < 8 h and clear fluids < 2 h; D, solid food ≥2 h < 6 h and clear fluids < 2 h; E, solid food < 2 h and clear fluids < 2 h

As shown in Table 3, a total of 1599 neonates were divided into 5 groups according to preoperative fasting of women. There were no statistical differences between groups, regardless of weight of neonates (*P* = 0.183) (Fig. 2a), gender (*P* > 0.05) (Fig. 2b), gestationalage (SGA) (*P* = 0.759) (Fig. 2c), gestationalage (LGA) (*P* = 0.791) (Fig. 2c) and the incidence of macrosomia (*P* > 0.05) (Fig. 2d). However, among all neonates, the proportion of LGA was much higher than that of SGA (Fig. 2c).

**Table 3:** Comparison of clinical data for neonates

Groups	Items	(N)	Birth weight (g)	Gender				SGA		LGA		Macrosomia	
	Fasting period		(M ± SD)	Baby boys		Baby girls							
				N	%	N	%	N	%	N	%	N	%
A	Solid food ≥8 h; clear fluids ≥6 h	501	3435 ± 858	250	49.9	251	50.1	25	5.0	155	31	55	11
B	Solid food ≥8 h; clear fluids ≥2 h < 6 h	297	3493 ± 480	149	50.2	148	49.8	15	5.2	87	29.4	35	11.8
C	Solid food ≥6 h < 8 h; clear fluids < 2 h	256	3478 ± 562	127	49.5	129	50.5	13	5.2	77	30.2	27	10.7
D	Solid food ≥2 h < 6 h; clear fluids < 2 h	150	3465 ± 653	75	50.0	75	50.0	7	4.8	46	30.8	18	12.1
E	Solid food < 2 h; clear fluids < 2 h	395	3488 ± 510	199	50.4	196	49.6	21	5.3	115	29.1	49	12.5
	Statistical value		2.012	3.225		0.359		0.235		0.169		0.989	
	*P* value		0.183	0.165		0.645		0.759		0.791		0.560	

*N* number, *BMI* body mass index, *M ± SD* mean ± standard deviation, *SGA* small for gestationalage, *LGA* large for gestationalage

### Influences of fasting time of women on incidence of adverse events to neonates

**Fig. 3:**
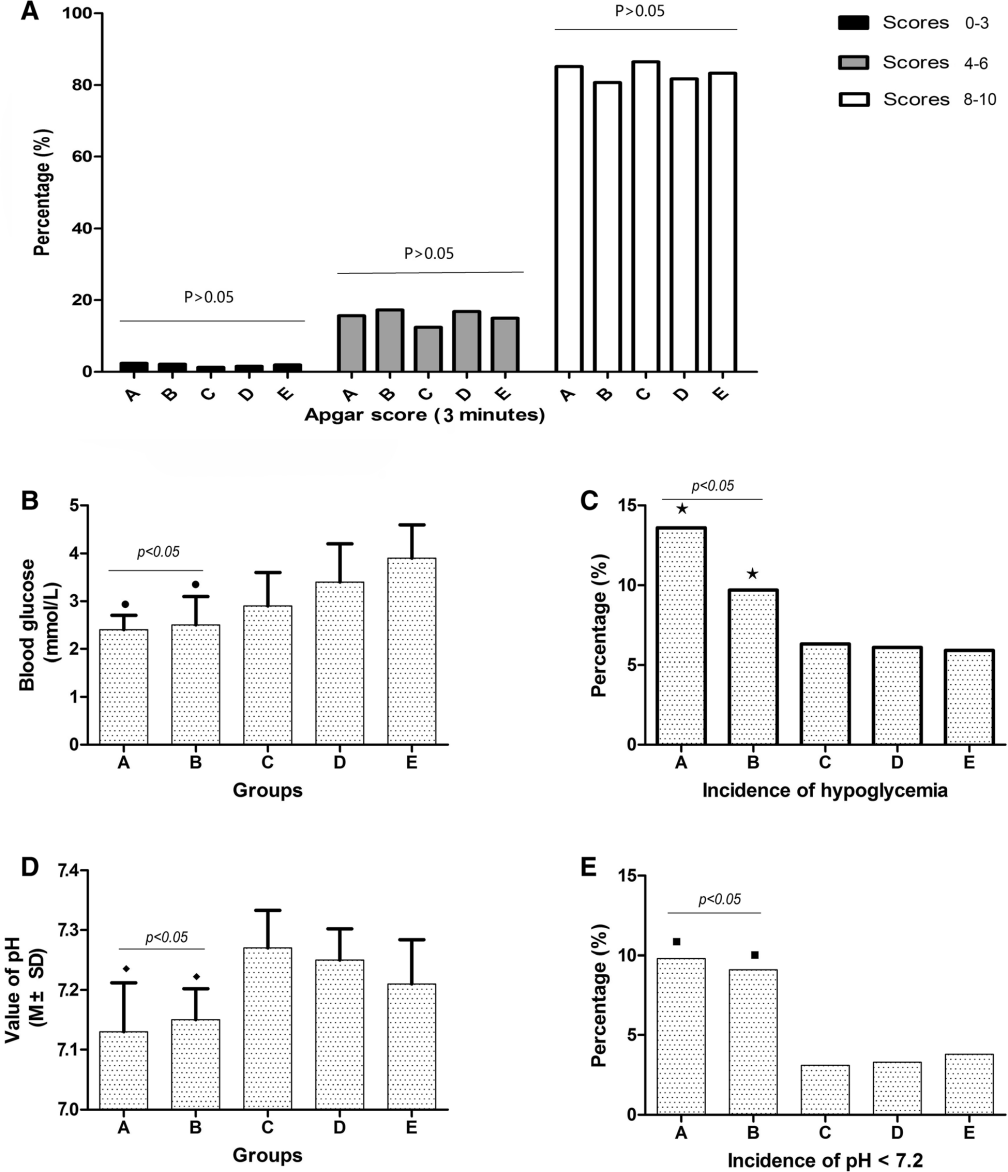
Influences of different fasting conditions of women on the health of neonates. **a**. The Apgar scores for neonates in 3 min between 5 groups did not show statistical difference (*P* < 0.05); **b**. blood glucose levels in group A and group B were significantly lower than those in C, D and E (*P* > 0.05); **c**. The incidence of hypoglycemia in group A and group B was significantly higher than that in group C, D and E (P < 0.05); **d**. The pH of neonatal umbilical arterial blood in group A and B displayed an obvious decrease compared with group C, D and E (P < 0.05); **e**. Incidence of pH < 7.2 in group A and group B were significantly higher than C, D and E (P < 0.05). Notes:A, solid food ≥8 h and clear fluids ≥6 h; B, solid food ≥8 h and clear fluids ≥2 h < 6 h; C, solid food ≥6 h < 8 h and clear fluids < 2 h; D, solid food ≥2 h < 6 h and clear fluids < 2 h; E, solid food < 2 h and clear fluids < 2 h

As shown in Table 4, the Apgar scores for neonates in 3 min between 5 groups did not show statistical difference (*P* > 0.05) (Fig. 3a). However, blood glucose levels in group A (2.4 ± 0.33 mmol/L) and group B (2.5 ± 0.62 mmol/L) were significantly lower than in group C (2.9 ± 0.75 mmol/L), D (3.4 ± 0.83 mmol/L) and E (3.9 ± 0.74 mmol/L) (*P* < 0.05) (Fig. 3b), and further suggesting that the incidence of hypoglycemia of neonates in group A (13.6%) and group B (9.7%) was significantly higher than that in group C (6.3%), D (6.1%) and E (5.9%) (*P* < 0.05) (Fig. 3c). Besides, the pH of neonatal umbilical arterial blood in group A (7.13 ± 0.82) and B (7.15 ± 0.52) were lower than that in group C (7.27 ± 0.63), D (7.25 ± 0.51) and E (7.21 ± 0.74) (P < 0.05) (Fig. 3d), thus the incidence of pH < 7.2 in Group A (9.8%) and group B (9.1%) were also significantly higher than C (3.1%), D (3.3%) and E (3.8%) (*P* < 0.05) (Fig. 3e).

**Table 4:** Incidence of adverse events for neonates

Groups	Items	Cases	Apgar score (3 min)			Blood glucose	Incidence of hypoglycemia		Value of pH	Incidence of pH < 7.2	
			Scores 0–3	Scores 4–6	Scores 7–10						
	Fasting period	(N)	N (%)	N (%)	N (%)	(mmol/L)	N	%	(M ± SD)	N	%
A	Solid food ≥8 h; clear fluids ≥6 h	501	12 (2.3)	78 (15.6)	411 (82.1)	2.4 ± 0.33^●^	68	13.6^★^	7.13 ± 0.82^◆^	49	9.8^■^
B	Solid food ≥8 h; clear fluids ≥2 h < 6 h	297	6 (2.1)	51 (17.2)	239 (80.7)	2.5 ± 0.62^●^	29	9.7^★^	7.15 ± 0.52^◆^	27	9.1^■^
C	Solid food ≥6 h < 8 h; clear fluids < 2 h	256	3 (1.2)	32 (12.4)	221 (86.4)	2.9 ± 0.75	16	6.3	7.27 ± 0.63	8	3.1
D	Solid food ≥2 h < 6 h; clear fluids < 2 h	150	2 (1.5)	25 (16.8)	123 (81.7)	3.4 ± 0.83	9	6.1	7.25 ± 0.51	5	3.3
E	Solid food < 2 h; clear fluids < 2 h	395	8 (1.9)	59 (14.9)	328 (83.2)	3.9 ± 0.74	23	5.9	7.21 ± 0.74	15	3.8
	*P* value		> 0.05	> 0.05	> 0.05	< 0.05	< 0.05		< 0.05	< 0.05	

*N* number, *M ± SD* mean ± standard deviation; ^●^, the blood glucose levels in groups A and B was significantly lower than that in group C, D and E; ^★^, the incidence of hypoglycemia in groups A and B was significantly higher than that in group C, D and E; ^◆^, the value of pH in groups A and B was significantly lower than that in group C, D and E; ^■^, the incidence of pH < 7.2 in groups A and B was significantly higher than that in group C, D and E

### Regression analysis for the effect of different fasting condition on neonates

Due to the different fasting conditions of women were related to neonates’ hypoglycemia and low-pH (*P* < 0.05), we further conducted a multi-factor regression analysis on different fasting times. As shown in Table 5, fasting conditions in Groups A (solid food ≥8 h and clear fluids ≥6 h) and B (solid food ≥8 h and clear fluids ≥2 h < 6 h) appeared to be risk factors for neonatal hypoglycaemia (for the condition of solid food ≥8 h and clear fluids ≥6 h: *P* < 0.001, OR = 9.158; for the condition of solid food ≥8 h and clear fluids ≥2 h < 6 h: *P* = 0.025, OR = 4.981) and acidosis (for the condition of solid food ≥8 h and clear fluids ≥6 h: *P* = 0.006, OR = 3.355; for the condition of solid food ≥8 h and clear fluids ≥2 h < 6 h: *P* = 0.008, OR = 3.368). Further analysis found that the effect of the fasting condition of solid food ≥8 h and clear fluids ≥6 h (OR = 9.158) on the hypoglycaemia was significantly higher than that of the condition of solid food ≥8 h and clear fluids ≥2 h < 6 h (OR = 4.981). However, for acidosis, the two did not have difference (OR = 3.355 versus 3.368).

**Table 5:** Regression analysis for the effect of different fasting conditions of women on hypoglycemia and pH < 7.2 of neonates

Influencing factors	B	SE	Word value	*P* value	OR	95% CI
Hypoglycemia						
Group A: Solid food ≥8 h; clear fluids ≥6 h	2.220	0.548	16.536	< 0.001	9.158	3.145 to 26.695
Group B: Solid food ≥8 h; clear fluids ≥2 h < 6 h	1.606	0.732	4.815	0.025	4.981	1.187 to 20.898
pH < 7.2						
Group A: Solid food ≥8 h; clear fluids ≥6 h	1.211	0.440	7.567	0.006	3.355	1.416 to 7.950
Group B: Solid food ≥8 h; clear fluids ≥2 h < 6 h	1.214	0.459	6.987	0.008	3.368	1.369 to 8.286

*B* regression coefficients, *SE* standard error, *OR* odds ratio, *CI* confidence interval

## Discussion

Intraoperative or postoperative vomiting has been considered as an important clinical issue affecting the safety of cesarean delivery [3]. Currently, the international guidelines for selective surgical fasting are 6 h of solid food and 2 h of clear fluids. These guidelines are based on the evidence that gastric emptying needs to be completed over these time periods because patients with shorter fasting periods have more stomach contents, which may result in increased risk of regurgitation and aspiration during anesthesia [14]. To understand the impact of different preoperative fasting conditions of women on neonates and themselves, we performed this study. In this study, a total of 1599 women were included and women and neonates were divided into 5 groups according to different fasting times of women: (A) solid food ≥8 h and clear fluids ≥6 h; (B) solid food ≥8 h and clear fluids ≥2 h < 6 h; (C) solid food ≥6 h < 8 h and clear fluids < 2 h; (D) solid food ≥2 h < 6 h and clear fluids < 2 h; and (E) solid food < 2 h and clear fluids < 2 h. Comparison of a series of clinical characteristic parameters, we found that the five groups of women had better clinical homogeneity and comparability.

Our study showed that whether on hypoglycemia, electrolyte imbalance or abdominal distension, the incidence rates of groups A and B (solid food > 8 h and clear fluids > 2 h at least) all obviously increased than other groups, which indicated that prolonged fasting is not conducive to the safety of the expectant mothers. Studies show that prolonged fasting leads to decline of glucose, amino acids and fatty acids, and may cause dehydration, electrolyte imbalance, malnutrition, nausea, hypothermia, fatigue and other adverse reactions [4, 7, 15]. In addition, we found that the incidence of vomiting (observation time is from the beginning of anesthesia to the end of surgery and recorded by anesthesiologists) in group C (solid food ≥6 h < 8 h and clear fluids < 2 h) was lower than other groups. It is reported that women receiving cesarean section after a full meal, vomiting, and even aspiration may be significantly increased [1, 4, 7]. Some opinions point out that prolonged fasting may lead to a lack of blood volume and supine hypotension due to epidural anesthesia, which may cause the incidence of vomiting [1, 3, 6, 8, 14]. Although the blood volume of women with short fasting (solid food < 6 h; clear fluids < 2 h at least) may not be affected by diet intake, the volume of the stomach increases, and the increase of the vagal excitability after the anesthesia of epidural nerve block will also increase the incidence of vomiting [1, 3, 6, 14].

The other goal of our study was to explore whether the different fasting times of women affect the health of neonates. When comparing the benchmark conditions, we found that the Apgar scores of neonates, birth weight, gender and the incidence rates of SGA, LGA and macrosomia in 5 groups did not have significant difference. However, the Apgar score is sometime performed with subjectivity to some extend, blood gas analysis of umbilical artery can directly reflect the acid-base levels of fetus [16]. Previous studies suggested that umbilical arterial blood gas and other indicators of diagnosis of asphyxia have good correlation and complementarity and can be one of the important objective evidence of diagnosis of asphyxia [17], and the diagnosis standard for acidosis using the umbilical cord blood pH < 7.2 was more sensitive than Apgar scores alone, and thus the missed diagnosis rate was reduced [17, 18]. In our study, the pH value of neonatal umbilical arterial blood in group A and B (solid food > 8 h and clear fluids > 2 h at least) displayed an obvious decrease compared to group C, D and E, which suggests that prolonged fasting of women increases the risk of acidosis in the neonates. Some of studies reported that prolonged fasting and incidence of vomiting will result in that fatty acids are oxidized in the liver, which further leads to ketonemia. Ketone bodies include acetone, beta-hydroxybutyric acid and acetic acid, and the latter two are organic acids, resulting in metabolic acidosis [19, 20].

Neonatal hypoglycemia may lead to apoptosis of nerve cells, residual visual impairment, cognitive impairment, occipital lobe epilepsy, cerebral palsy and other sequelae [21]. Previous studies show that blood glucose was negatively correlated with gestational age and birth weight and was positively correlated with the degree of infection, and some other factors such as delivery mode, blood glucose in pregnant women, and the combination of ketosis in women are also related to the blood glucose level of the neonates [4, 21–23]. Our study showed that the incidence of hypoglycemia in group A and group B (solid food > 8 h and clear fluids > 2 h at least) were significantly higher than that in other groups, suggesting that the potential risk factors for long-term fasting can not be ignored in clinical practice. In our study, the proportion of hypoglycemia neonates having clinical performance only accounted for 10.5%, and the clinical symptoms mainly included cyanosis of oral and/or the whole body, poor response, moans, shortness of breath and breathing difficulties. Through a regression analysis, we found that the fasting conditions in groups A (solid food ≥8 h; clear fluids ≥6 h) and B (solid food ≥8 h; clear fluids ≥2 h < 6 h) appeared to be risk factors for neonatal hypoglycaemia and acidosis. Although pH < 7.2 can not be used as an absolute risk factor for the diagnosis of neonatal asphyxia, it still needs to be followed closely to observe the possible risks.

However, there are still some shortcomings in this study. Firstly, this is a retrospective study, not a randomized controlled prospective study, and therefore there may be inherent bias within group allocation. Secondly, the sample size assigned to each group is not very balanced and may affect statistical performance to some extent. Thirdly, the observation indicators of this study are limited. Hence, further randomized controlled clinical interventional studies should be done.

## Conclusion

The preoperative fasting of solid food ≥6 h to < 8 h and clear fluids < 2 h had potential advantages to both of women and neonates, which decreased the incidence rate of neonatal hypoglycemia and acidosis as well as the incidence of vomiting of women.

## Abbreviations

ASA: American Academy of Anesthesiologists
BMI: Body mass index
LGA: Large for gestationalage
SGA: Large for gestationalage
WHO: World Health Organization
